# Supplementary material for: Development of a Short-Cut Combined Magnetic Coagulation–Sequence Batch Membrane Bioreactor for Swine Wastewater Treatment
Source: Membranes (Basel). 2021 Jan 23;11(2):83. doi: 10.3390/membranes11020083 (PMC7911319; doi:10.3390/membranes11020083)
Supplement: Supplementary file 1 [file membranes-11-00083-s001.pdf]

Supplemental materials

Development of a Short-Cut Combined Magnetic Coagulation–Sequence Batch Membrane Bioreactor for Swine Wastewater Treatment

Yanlin Chen [1,2,3], Qianwen Sui [1,2], Dawei Yu [1,2], Libing Zheng [1,2], Meixue Chen [1,2] and Tharindu Ritigala [1,2,3], Yuansong Wei [1,2,3,4,*]

[1] State Key Joint Laboratory of Environment Simulation and Pollution Control, Research Center for Eco-Environmental Sciences, Chinese Academy of Sciences, Beijing 100085, China; ylchen_st@rcees.ac.cn (Y.C.); qwsui@rcees.ac.cn (Q.S.); dwyu@rcees.ac.cn (D.Y.); lbzheng@rcees.ac.cn (L.Z.); mxchen@rcees.ac.cn (M.C.); tharindu_st@rcees.ac.cn (T.R.)

[2] Laboratory of Water Pollution Control Technology, Research Center for Eco-Environmental Sciences, Chinese Academy of Sciences, Beijing 100085, China;

[3] University of Chinese Academy of Sciences, Beijing 100049, China;

[4] Institute of Energy, Jiangxi Academy of Sciences, Nanchang 330029, China

* Correspondence: yswei@rcees.ac.cn.

Eq. S1: Activity of ammonia oxidation bacteria

$$K_{AOB} = \frac{K}{MLSS} \tag{S1}$$

Where K is the degradation rate constant of ammonia nitrogen, mgN/(L·h); MLSS is the mixed liquor suspended solids in SMBR, mg/L.

Eq. S2: Activity of nitrite oxidation bacteria

$$K_{NOB} = \frac{K}{MLSS} \tag{S2}$$

Where K is the formation rate constant of nitrate, mgN/(L·h); MLSS is the mixed liquor suspended solids in SMBR, mg/L.

Eq. S3: Free ammonia (FA)

$$\mathrm{FA} = \frac{17}{14} \times \frac{([NH_3-N]+[NH_4-N])\times 10^{pH}}{e^{\frac{6344}{273+t}}+10^{pH}} \tag{S3}$$

Where FA is the free ammonia concentration, mg-NH_3/L; ([NH_3-N]+[NH_4^+-N]) is the total ammonium nitrogen in the reactor, mg/L; t is the temperature, °C; and pH is the pH value.

Table.S1 Performance of magnetic coagulation pretreatment of swine wastewater at different stages

Stage	PO_3^--P			C/N	
	Influent(mg/L)	Effluent(mg/L)	Removal efficiency (%)	Influent	Effluent
I	114.3 ±8.6	-	-	8.7 ±1.7	-
II	140.2 ±15.2	43.4 ±2.7	68.8 ±2.7	8.1 ±1.3	5.4 ±1.4
III	134.4 ±13.9	30.5 ±3.7	77.3 ±4.9	11.2 ±2.1	4.1 ±1.6

Table S2 Operating parameters and performance of the SMBR at different stages (mg/L)

Stage	HRT (d)	Load / kg(kgVSS·d)$^-$			TN			TP		
		COD	TN	NH_4^+-N	Inf	Eff	Remove rate (%)	Inf	Eff	Remove rate (%)
I	5.0	0.4 ±0.04	0.049 ±0.008	0.042 ±0.005	1097.0 ±184.5	38.0 ±4.4	97.0 ±0.5	132.4 ±5.8	125.3 ±8.3	5.5 ±1.2
II	4.7	0.3 ±0.09	0.067 ±0.025	0.057 ±0.014	1422.7 ±534.8	36.9 ±7.2	97.1 ±0.9	52.2 ±2.7	49.2 ±3.9	5.8 ±0.4
III	4.3	0.2 ±0.12	0.062 ±0.015	0.050 ±0.009	1201.4 ±297.5	32.3 ±5.8	97.3 ±0.4	35.8 ±5.1	33.2 ±2.8	6.4 ±0.7

Table S3 Performance comparison of the combined magnetic coagulation-SMBR process treating swine wastewater (mg/L)

Reactor	HRT (d)	COD			NH_4^+-N			TN			TP			Reference
		Influent	Effluent	Removal efficiency (%)	Influent	Effluent	Removal efficiency (%)	Influent	Effluent	Removal efficiency (%)	Influent	Effluent	Removal efficiency (%)	
SMBR	6.0	7046.27	327.34	95.3	811.71	10.19	98.7	1042.54	75.39	92.8	-	-	-	[1]
SMSBR[a]	4.0	4400	132	97.0	-	-	-	1300	143	89.0	344.0	68.8	80.0	[2]
SMBR in Stage Ⅰ	5.0	9227.2	335.9	96.4	943.4	8.6	99.1	1097.0	38.0	96.5	132.4	125.3	5.4	This study
MC-SMBR process in Stage II	4.7	10141.3	401.6	96.0	1245.7	12.4	99.0	1514.9	36.9	97.6	159.1	49.2	69.1	This study
MC-SMBR process in Stage III	4.3	11507.3	340.3	97.0	1031.3	7.1	99.3	1201.5	32.3	97.3	152.4	33.2	78.2	This study

[a] SMSBR: submerged membrane sequencing batch reactor; MC: magnetic coagulation

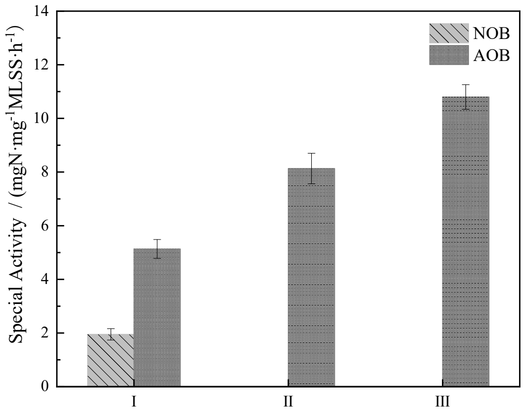

Figure S1. Activity of AOB&NOB in the SMBR at different stages

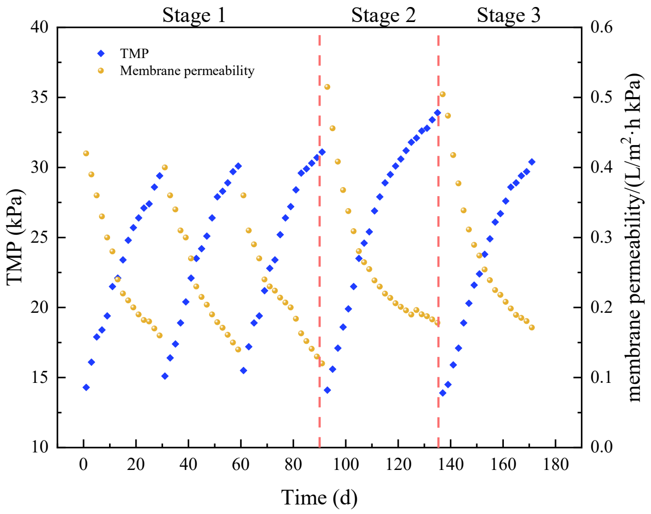

Figure S2. TMP and membrane flux in SMBR at different stages

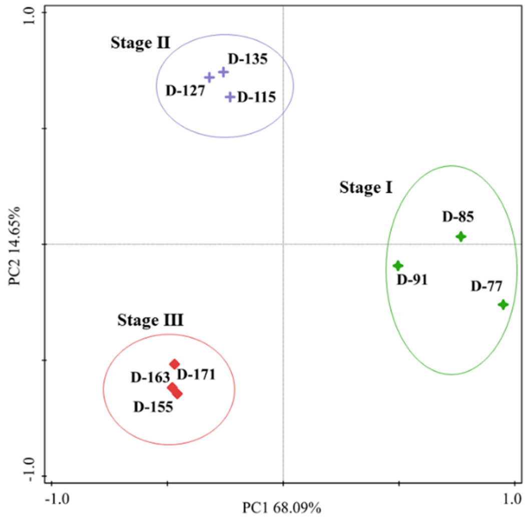

Figure S3. Principle component analysis of the microbial community in the SMBR

Reference

[1] Sui, Q., Jiang, C., Yu, D., Chen, M., Zhang, J., Wang, Y., Wei, Y. 2018. Performance of a sequencing-batch membrane bioreactor (SMBR) with an automatic control strategy treating high-strength swine wastewater. *Journal of Hazardous Materials*, **342**(15), 210-219.

[2] Han, Z., Chen, S., Lin, X., Yu, H., Duan, L.A., Ye, Z., Jia, Y., Zhu, S., Liu, D. 2017. Performance and membrane fouling of a step-fed submerged membrane sequencing batch reactor treating swine biogas digestion slurry. *Journal of Environmental Sciences And Health, Part A*, 1-8.
